# Longitudinal assessment of intraocular pressure in the 5xFAD mouse model of Alzheimer's disease

**DOI:** 10.3389/fneur.2026.1858031

**Published:** 2026-07-21

**Authors:** Raz Rubinshtein, Basel Obied, Talal Salti, Andres I. König, Alon Zahavi, Nitza Goldenberg-Cohen

**Affiliations:** 1 Ophthalmology Department, The Krieger Eye Research Laboratory, Bruce and Ruth Faculty of Medicine, Technion-Institute of Technology, Haifa, Israel; 2 Department of Ophthalmology, Bnai-Zion Medical Center, Haifa, Israel; 3 Ophthalmology Department, School of Neurobiology, Biochemistry and Biophysics, Tel-Aviv University, Tel Aviv, Israel; 4 Department of Ophthalmology and Laboratory of Eye Research, Rabin Medical Center, Petach Tikva, Israel; 5 Ophthalmology Department, Gray Faculty of Medical and Health Sciences, Tel Aviv University, Tel Aviv, Israel

**Keywords:** 5xFAD transgenic mice, Alzheimer's disease, glaucoma, intraocular pressure, mouse model

## Abstract

**Objectives:**

Glaucoma and Alzheimer's disease (AD) are major neurodegenerative disorders with increasing evidence of shared pathogenic pathways. Glaucoma involves progressive optic nerve degeneration and irreversible vision loss, often associated with elevated intraocular pressure (IOP) but also occurring independently of it. AD, the leading cause of dementia, results in progressive cognitive and functional decline, with vision disturbances including visual field defects. Epidemiological studies report higher co-prevalence of glaucoma and AD in older adults. This study longitudinally assessed IOP in a transgenic AD mouse model to determine whether AD-related amyloid pathology inherently drives alterations in ocular pressure.

**Methods:**

Ten young (25 weeks old; 9 males, 1 female) and fifteen aged 5xFAD (57–60 weeks old; 5 males and 10 females) transgenic mice, a well-established amyloidogenic model of AD, were examined. Age-matched control groups included ten young wild type (WT) mice (9 males and 1 female) and fourteen aged WT mice (7 males and 7 females). IOP was measured repeatedly without anesthesia using a rebound tonometer (Icare Tonolab) calibrated for mice. Four IOP measurement sessions were performed at days 1, 36, 55, and 77, with all measurements conducted during midday hours (11:00–14:00) to minimize circadian variability.

**Results:**

IOP remained stable across most groups and time points. Aged 5xFAD mice exhibited transient, statistically significant fluctuations, characterized by an initial decrease at day 36 followed by a return to baseline levels. Age-matched WT mice showed no significant longitudinal changes. When comparing between groups, the only significant difference was observed at day 36, where aged 5xFAD mice demonstrated significantly lower IOP than aged WT controls.

**Conclusions:**

5xFAD mice did not exhibit sustained IOP elevation compared with WT controls, with aged animals displaying only transient fluctuations that likely reflect physiological or measurement variability. These results suggest that amyloid-driven pathology in this model is not accompanied by chronic ocular hypertension. Consequently, our findings support the hypothesis that visual dysfunction in AD models may occur independently of elevated intraocular pressure, though the specific overlapping mechanisms between AD and glaucoma warrant cautious interpretation and further investigation.

## 1 Introduction

Alzheimer's disease (AD) affects more than 7 million Americans and over 55 million people worldwide. About 1 in 9 people age 65 and older (11%) has AD, representing one of the most devastating neurodegenerative disorders and a major global public health challenge (1). It is estimated that by 2050, approximately 13.8 million people in the United States will be living with AD (2). AD is characterized by a progressive decline in learning, memory, and executive functions (2). In addition to cognitive and behavioral deficits, vision disturbances have been reported in the early stages of AD, well before the diagnosis is clearly established (3). Optic neuropathy was also associated with AD, but the underlying mechanisms remain poorly defined (4, 5). The pathophysiology of AD is not fully understood. Proposed pathways include amyloid β (Aβ) deposition, tau pathology, impaired cholinergic and glutamatergic signaling, and disturbances in oxidative stress and calcium homeostasis (6).

Glaucoma is a progressive neurodegenerative optic neuropathy and the leading cause of irreversible blindness worldwide (7). While it is classically characterized by optic nerve head cupping and associated visual field loss, glaucoma has also been associated with structural and functional brain changes (8–10). Elevated intraocular pressure (IOP) is considered the primary modifiable risk factor; however, disease progression often continues despite IOP reduction, suggesting the involvement of additional pathophysiologic mechanisms beyond increased IOP (11, 12). A possible relationship between glaucoma and neurodegenerative diseases such as AD has been previously suggested (13–15). However, some studies have not found an increased risk of AD in patients with glaucoma (13, 16). Furthermore, the apolipoprotein E4 (APOE4) allele, which is associated with an increased risk of AD, has been reported to be associated with a decreased risk of glaucoma (17). Indeed, mounting evidence suggests that the neurodegenerative links between AD and glaucoma, including amyloid deposition, neuroinflammation, and mitochondrial dysfunction, are largely IOP-independent (14–16).

5xFAD mice are a well-established transgenic model of AD, carrying five familial AD mutations that drive rapid Aβ deposition. They develop early Aβ pathology by 2 months, followed by neuroinflammation, synaptic dysfunction, and cognitive decline by 4–6 months, which parallels key features of human AD (18–21). Importantly, Aβ accumulation also occurs in the retina, where 5xFAD mice show progressive thinning of the retinal nerve fiber layer and dysfunction of retinal ganglion cells (RGCs) and photoreceptors (22–24). These changes apparently occur independently of IOP dysregulation or glaucomatous optic neuropathy (22–24). In this study, we measured IOP in aging transgenic 5xFAD mice to examine whether AD-related neurodegeneration and amyloid accumulation inherently drive sustained alterations in ocular pressure.

## 2 Materials and methods

### 2.1 Study animals

Transgenic 5xFAD mice and age-matched wild-type (WT) controls were used in this study. At study onset, 10 young 5xFAD mice (25 weeks old; 9 males and 1 female), 15 old 5xFAD mice (57–60 weeks old; 5 males and 10 females), 10 young WT mice (25 weeks old; 9 males and 1 female), and 14 old WT mice (57–60 weeks old; 7 males and 7 females) were included. Due to planned euthanasia to harvest tissues for a parallel independent study (in accordance with the 3Rs ethical principle of reduction), a subset of animals did not complete all four time points. Consequently, the final day of analyses included 10 young 5xFAD, 13 aged 5xFAD, 6 young WT, and 9 aged WT mice. No animals or data points were excluded due to adverse events, technical failures, or predefined exclusion criteria, and all valid IOP data collected prior to euthanasia were retained for analysis. Animals were maintained under standard housing conditions (12 h light/12 h dark cycle, dry chow and water provided *ad libitum*). All procedures were approved by the Institutional Animal Care and Use Committee (approval number IL0212025).

### 2.2 Experimental design

A total of four IOP measurement sessions were conducted on days 1, 36, 55, and 77. All measurements were performed during the midday hours (11:00–14:00) to minimize circadian variability.

### 2.3 IOP measurements

IOP was measured using iCare TONOLAB^®^. During each measurement, one researcher gently restrained the mouse by hand in a calm and supportive manner, without applying pressure to the body or head, while a second researcher operated the device. For each eye, three independent readings were recorded. Each reading represents the device's internal quality-controlled average of six rapid automated probes. These three readings were then averaged to obtain a single mean IOP per eye. Finally, to prevent clustering bias and define the individual animal as the unit of analysis, the values from both eyes were averaged to yield a single final IOP value per mouse per session. Measurements were performed without anesthesia to avoid confounding effects on IOP. To maximize technical consistency and minimize handling-induced variability, all measurement sessions were performed by the exact same team of two investigators, with one strictly responsible for animal restraint and the second operating the device. Animals were evaluated in a consistent, structured sequence during each session. Investigators were not blinded to genotype or age group during testing, and no proactive data exclusions were applied to the surviving cohorts.

### 2.4 Statistical analysis

Statistical analyses were performed using R (R Core Team. R: A Language and Environment for Statistical Computing. R Foundation for Statistical Computing, Vienna, Austria; 2025. https://www.R-project.org). The individual animal served as the unit of analysis. For each measurement session, IOP values from the left and right eyes were averaged to obtain a single IOP value per mouse.

Because IOP measurements were obtained repeatedly from the same animals over time and some animals did not complete all follow-up visits due to planned euthanasia, longitudinal analyses were performed using a linear mixed-effects model. Genotype (5xFAD vs. WT), age group (young vs. aged), timepoint (days 1, 36, 55, and 77), and their interactions were included as fixed effects, while animal identity was included as a random intercept to account for repeated measurements within animals.

Model assumptions were assessed by inspection of residual quantile-quantile (Q-Q) plots and formal testing using the Shapiro-Wilk test for normality of residuals. Homogeneity of variance was evaluated using Levene's test. Sensitivity analyses, including log-transformed models and evaluation of influential observations, were performed to assess the robustness of the findings.

Data are presented as mean ± standard deviation (SD). Ninety-five percent confidence intervals (95% CIs) were calculated using Student's t distribution. Effect sizes are reported as Hedges' g with 95% confidence intervals for pairwise comparisons and partial η^2^ with 90% confidence intervals for fixed effects. Statistical significance was defined as *p* < 0.05.

## 3 Results

IOP was measured longitudinally in young and aged 5xFAD mice and age-matched WT controls. Overall, IOP values remained relatively stable across most groups and time points, with only a limited number of statistically significant changes detected.

Linear mixed-effects modeling demonstrated no overall effect of genotype on IOP (*p* = 0.954, partial η^2^ = 0.000) and no significant effect of age group (*p* = 0.604, partial η^2^ = 0.007). A significant effect of timepoint was observed (*p* = 0.001, partial η^2^ = 0.128), and a significant genotype-by-age interaction was detected (*p* = 0.020, partial η^2^ = 0.128), whereas the genotype-by-time interaction did not reach statistical significance (*p* = 0.069, partial η^2^ = 0.060).

**Figure 1:**
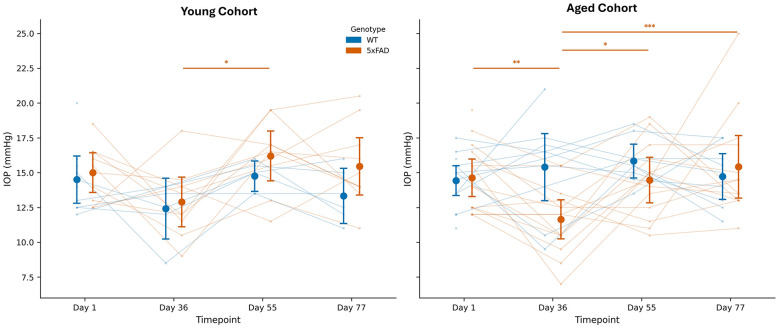
Longitudinal intraocular pressure measurements in young and aged WT and 5xFAD mice. Mean ± SD IOP values are shown at Days 1, 36, 55, and 77. Blue indicates WT mice and orange indicates 5xFAD mice. Faint lines represent individual animals. Significant within-group longitudinal comparisons are indicated by horizontal brackets (*0.05 < *p* < 0.01, ** 0.01 < *p* < 0.001, *** *p* < 0.001). No significant longitudinal changes were observed in WT mice. The only significant between-group difference occurred at Day 36 in the aged cohort, where 5xFAD mice had lower IOP than age-matched WT controls (*p* = 0.0022).

In the aged 5xFAD group, IOP showed a significant decrease between day 1 and day 36 (14.63 ± 2.43 mmHg, 11.64 ± 2.43 mmHg, respectively, *p* = 0.0086), followed by a significant increase between day 36 and day 55 (11.64 ± 2.43 mmHg, 14.46 ± 2.7 mmHg, respectively, *p* = 0.0207) and between day 36 and day 77 (11.64 ± 2.43 mmHg, 15.42 ± 3.72 mmHg, respectively, *p* = 0.0008). In the young 5xFAD group, a modest but statistically significant increase was observed between day 36 and day 55 (12.9 ± 2.49 mmHg, 16.2 ± 2.5 mmHg, respectively, *p* = 0.0193). No significant longitudinal changes were found in either WT control group. The IOP values are presented in Table 1 and Figure 1.

**Table 1:** Longitudinal intraocular pressure (IOP) measurements in wild-type (WT) and 5xFAD transgenic mice at multiple time points.

Time point	Group	Number	Mean IOP (mmHg)	SD	95% CI (Low, High)
Day 1	5xFAD aged	15	14.63	2.43	13.29, 15.98
	5xFAD young	10	15	1.99	13.58, 16.42
	WT aged	14	14.43	1.85	13.36, 15.5
	WT young	10	14.5	2.37	12.81, 16.19
Day 36	5xFAD aged	14	11.64	2.43	10.24, 13.05
	5xFAD young	10	12.9	2.49	11.12, 14.68
	WT aged	10	15.4	3.36	12.99, 17.81
	WT young	6	12.42	2.08	10.23, 14.6
Day 55	5xFAD aged	13	14.46	2.7	12.83, 16.1
	5xFAD young	10	16.2	2.5	14.41, 17.99
	WT aged	9	15.83	1.58	14.62, 17.05
	WT young	6	14.75	1.04	13.66, 15.84
Day 77	5xFAD aged	13	15.42	3.72	13.18, 17.67
	5xFAD young	10	15.45	2.88	13.39, 17.51
	WT aged	9	14.72	2.14	13.08, 16.37
	WT young	6	13.33	1.89	11.35, 15.32

Separate panels show young and aged cohorts, allowing comparison of IOP trajectories across age and genotype.

When comparing groups at individual time points, most pairwise comparisons showed no significant differences. The only exception was observed on day 36, when the aged 5xFAD group had significantly lower IOP than aged WT mice (11.64 ± 2.43 mmHg vs. 15.40 ± 3.36 mmHg, respectively; *p* = 0.0022; Hedges' g = −1.47, 95% CI −2.36 to −0.59). Exact *p*-values for all between-group comparisons are provided in Supplementary Table S1.

Taken together, these findings indicate that while IOP remained largely stable across groups, aging 5xFAD mice exhibited transient but statistically significant fluctuations in IOP that were not observed in WT controls.

## 4 Discussion

In this study, we repeatedly assessed IOP in 5xFAD mice and WT controls in young and aged cohorts. Overall, IOP remained stable within the normal range across all groups, with limited, transient fluctuations detected only in 5xFAD mice, particularly in the aged group. Notably, WT controls did not show significant changes over time in IOP or internal variability within the group. In both groups, age was not associated with increased IOP. All IOP exams were performed without anesthesia. The transient IOP fluctuations observed in the aged 5xFAD group must be interpreted cautiously, as they likely stem from a combination of measurement variability, biological variability, and sample size limitations, rather than true pathological ocular hypertension. First, the relatively modest sample size in the aging cohorts may amplify the statistical impact of transient physiological shifts. Second, awake IOP measurements are inherently susceptible to handling-induced measurement variability. Consequently, the observed IOP fluctuations likely reflect an amplified stress response and a failure to adapt to repeated awake examinations, rather than a sustained glaucomatous trait. However, we cannot exclude the possibility of concurrent biomechanical or physiological shifts, such as age- or disease-related changes in corneal properties, aqueous humor dynamics, or translaminar pressure gradients. Because these parameters were not directly measured, such mechanisms remain strictly hypothetical. Importantly, no sustained elevated IOP peak was detected in any of the 5xFAD mice. These findings align with the broader view that, unlike in classical glaucoma models, AD pathology alone may not consistently or sustainably elevate IOP.

As a neurodegenerative condition, glaucoma is characterized by progressive optic nerve damage associated with RGC loss and corresponding visual field defects. Although the underlying etiology remains incompletely elucidated, the association between AD and glaucoma is well supported by epidemiological evidence. Both conditions are prevalent neurodegenerative disorders with substantial global health impact, characterized by progressive neuronal loss and irreversible functional decline (7, 25–27). Large population-based cohort studies, including national datasets from Sweden and Korea, have demonstrated that individuals with glaucoma have an increased risk of developing AD and other dementias, particularly at older ages (28, 29). Meta-analyses further corroborate this association, indicating that glaucoma is linked to a higher risk of all-cause dementia and AD, though not vascular dementia (30).

It is not clear whether there is an increased prevalence of glaucoma among AD patients (31). It was previously suggested that both glaucoma and AD cause RGC loss. The discovery of deposition of Aβ and tau proteins in the retina was associated with a possible mechanism underlying glaucoma (31). However, although this theory seems reasonable, as the accumulation of protein might occlude the trabecular meshwork and increase IOP, there is no evidence of deposition in the anterior chamber or trabecular meshwork. The possible association between protein accumulation and increased intraocular or intracranial pressure remains inconclusive. There is no evidence of changes in gradients of the trans-lamina cribrosa or in vascular autoregulation factors that contribute to AD-related and glaucoma visual loss (31). In this study, we showed that IOP is not increased in 5xFAD mice with proven Aβ accumulation (32, 33).

Several studies have suggested shared genetic susceptibility between glaucoma and AD, but the underlying associations remain incompletely defined (34). The apolipoprotein E (APOE) gene has been a major focus of investigation, as specific alleles such as APOE ε4 are associated with an increased risk of AD and have also been implicated in glaucoma (8, 35). Mechanistically, both diseases share overlapping pathogenic features, including extracellular Aβ accumulation, tauopathy, neuroinflammation, and vascular dysfunction (32, 36). Aβ deposits and abnormal tau isoforms have been identified in the retinas of AD patients and animal models, with RGC degeneration and nerve fiber layer (RNFL) thinning observed in both conditions (32, 33). The ocular glymphatic system appears to facilitate the transport of Aβ from the brain to the eye, contributing to retinal degeneration in AD and potentially linking cerebral and ocular pathology (37, 38). Additionally, autoimmune phenomena, such as agonistic autoantibodies targeting the β2-adrenergic receptor, have been implicated in both AD and glaucoma, suggesting a shared adrenergic dysregulation (39).

Advances in retinal imaging and proteomics have enabled the detection of AD-specific pathology in the eye, supporting the retina as a noninvasive window for early diagnosis and disease monitoring (32, 40, 41). The correlation between retinal and cerebral Aβ burden, as demonstrated in transgenic mouse models, underscores the systemic nature of amyloid pathology and highlights the potential of ocular biomarkers for risk stratification and therapeutic targeting (24, 41). Our results, therefore, suggest that retinal alterations reported in this model are unlikely to be driven by chronic ocular hypertension. Instead, they suggest that any pathological overlap between Alzheimer's disease and glaucoma is more likely to involve pressure-independent, NTG-like mechanisms. Plausible explanatory frameworks for such neurodegeneration include vascular dysregulation, alterations in the CSF-related translaminar pressure gradient, impaired perivascular or glymphatic clearance, and neuroinflammatory pathways. While these processes were not directly evaluated in the present study, they provide a broader theoretical context for understanding progressive retinal ganglion cell loss independent of elevated intraocular pressure. For instance, recent evidence highlighting shared microRNA expression profiles between AD and normal-tension glaucoma further supports that these neurodegenerative links are driven by alternative molecular pathways rather than elevated intraocular pressure (42).

Recent investigations into ocular physiology in 5xFAD mice have primarily focused on retinal structure, function, and vascular metrics, with limited direct assessment of IOP. Lynn et al. (24). and Lim et al. (43) demonstrated that 5xFAD mice exhibit early and progressive retinal dysfunction, including photoreceptor and ganglion cell impairment, as well as structural changes such as thinning of the RNFL and thickening of the inner plexiform layer, but neither report included assessments of IOP. Similarly, Matei et al. identified alterations in venous diameter and blood velocity in 5xFAD mice. However, their study did not evaluate IOP, focusing instead on hemodynamics and oxygen delivery (44). These findings are consistent with our results and support the interpretation that amyloid-driven retinal pathology in 5xFAD mice may occur independently of IOP elevation.

Visual field abnormalities have been reported in both glaucoma and patients with AD. In AD patients, these likely reflect neurodegenerative and synaptic alterations related to Aβ pathology (45, 46). Studies using standard automated perimetry and frequency-doubling technology have demonstrated visual field deficits in patients with AD compared with age-matched controls, with severity correlating with cognitive decline. Because both glaucoma and AD are prevalent in older populations, these visual field changes may mimic glaucomatous defects, complicating clinical differentiation. Some studies have reported medial temporal lobe atrophy associated with visual field abnormalities in AD (47), although the underlying mechanisms remain incompletely understood.

It should be noted that some visual field defects reported in AD occur in patients with Posterior Cortical Atrophy (PCA), a visual-predominant syndrome within the AD spectrum characterized by degeneration of posterior cortical regions. In contrast to glaucomatous visual field loss, visual field defects in PCA are frequently homonymous and reflect post-chiasmal involvement rather than retinal or optic nerve pathology. Therefore, when evaluating visual field abnormalities in patients with AD, it is important to distinguish homonymous cortical defects from defects arising from ocular disease, including glaucoma, before attributing visual dysfunction to retinal neurodegeneration or glaucomatous damage (48).

Methodological considerations also play an important role in IOP research. Advances such as the non-invasive fixation device described by Guo et al. (49) have improved the accuracy of IOP measurement in awake mice, complementing earlier studies showing that IOP is strongly influenced by strain, age, and anesthesia (50–53). Our study extends this literature by directly measuring IOP in 5xFAD mice under standardized conditions and without anesthesia, minimizing potential confounding variables. On the other hand, without anesthesia, behavioral, cognitive, and memory decline may affect the mouse stress response to repeated exams, placing the transgenic mice at presumably higher stress levels and thus affecting IOP measurements. It should be noted that in our study age did not affect IOP measurements.

This study has several limitations. First, although RGC degeneration has been reported in 5xFAD mice in previous studies, the present work focused specifically on assessing IOP and did not include direct structural or functional retinal measurements. Second, there was a reduction in sample size over the course of the longitudinal study due to planned euthanasia for tissue collection as part of a parallel study. This reduced the number of animals available at later time points and may have limited statistical power to detect subtle longitudinal changes in IOP. Nevertheless, the internal variability within the surviving WT mice remained consistently low, suggesting that they still provided a stable and reliable physiological baseline for comparison. Third, investigators were not blinded to the genotype or age groups during IOP testing, which introduces a potential risk of operator bias inherent to awake rebound tonometry. However, this risk was mitigated by maintaining a strict, two-person protocol where the same individuals performed identical roles across all experimental sessions to maximize reproducibility. Fourth, we note the differing sex distribution between our young and aged cohorts. While sex is known to modulate certain pathological features in the 5xFAD model, the limited number of females in the young groups precluded a reliable stratified analysis or the inclusion of sex as a statistical covariate. Nonetheless, the highly consistent baseline values observed across sessions suggest that our findings capture a robust physiological profile of IOP across these age groups. Fifth, rebound tonometry estimates IOP based on probe deceleration and rebound characteristics, making it potentially susceptible to variations in corneal biomechanics, such as central corneal thickness or corneal stiffness. Because these parameters were not measured in the current study, we cannot fully exclude the possibility that transient fluctuations, such as the lower mean IOP recorded at day 36, partly reflect differences in corneal properties rather than true changes in hydrostatic intraocular pressure. Sixth, our starting cohort of 25-week-old mice represents an established stage of amyloid pathology in the 5xFAD model, rather than a true pre-pathological baseline. Because amyloid deposition typically begins much earlier in this model, the absence of a younger cohort (e.g., 6–8 weeks of age) limits our ability to evaluate whether the absolute initial onset of amyloid accumulation and early neuroinflammation are associated with transient changes in IOP. Seventh, the 5xFAD model is primarily amyloid-driven and does not fully reproduce the tau pathology characteristic of human AD. Therefore, the absence of sustained IOP elevation in this specific model does not exclude the possibility of tau-mediated or other pressure-independent pathways contributing to a shared pathogenesis between AD and glaucoma. In addition, IOP measurements were performed without anesthesia to avoid the known effects of anesthesia on ocular pressure; however, as noted, this approach may introduce variability due to animal handling and stress. Finally, the transient IOP differences observed in the aged 5xFAD group were not sustained across time points and may reflect physiological or measurement variability rather than pathological ocular hypertension.

Taken together, ourfindings demonstrate that amyloid-driven pathology in the 5xFAD model is not accompanied by chronic ocular hypertension. These observations support the hypothesis that visual dysfunction in AD may resemble glaucomatous damage but likely occurs independently of elevated intraocular pressure. As such, while our data highlight an IOP-independent relationship, interpretations regarding the specific overlapping mechanisms between AD and glaucoma should be made cautiously and warrant further investigation. Recent research has extensively explored the multifactorial nature of visual field deficits in AD, highlighting the roles of retinal amyloid accumulation alongside central nervous system changes (54–57). While other experimental models of AD may exhibit different ocular pressure profiles, our data indicate that in the 5xFAD model, potential Aβ-associated retinal pathology is not accompanied by sustained IOP elevation in either young or aged animals. Across all time points examined, IOP remained within a comparable range to that of WT controls. These findings suggest that the optic nerve and RGC vulnerability observed in AD models can occur independently of IOP elevation and do not constitute a pressure-dependent risk factor for glaucomatous optic neuropathy. Potential interactions between amyloid pathology and additional ocular or systemic stressors may further modulate neurodegeneration; however, such mechanisms remain outside the scope of the present study.

## Data Availability

The original contributions presented in the study are included in the article/Supplementary material, further inquiries can be directed to the corresponding author.
