# Supplementary material for: Longitudinal assessment of intraocular pressure in the 5xFAD mouse model of Alzheimer's disease
Source: Front Neurol. 2026 Jul 21;17:1858031. doi: 10.3389/fneur.2026.1858031 (PMC13433196; doi:10.3389/fneur.2026.1858031)
Supplement: Supplementary file 1 [file Table_1.docx]

| Timepoint | Comparison | Mean Difference (mmHg) | 95% CI Lower | 95% CI Upper | df | t | Unadjusted p Value | Adjusted p Value (Sidak) | Hedges' g | Hedges' g 95% CI Lower | Hedges' g 95% CI Upper |
| --- | --- | --- | --- | --- | --- | --- | --- | --- | --- | --- | --- |
| Day1 | 5xFAD Aged vs WT Aged | 0.205 | -2.369 | 2.779 | 147.7 | 0.22 | 0.82611 | 1 | 0.082 | -0.626 | 0.79 |
| Day1 | 5xFAD Young vs WT Young | 0.5 | -2.597 | 3.597 | 147.7 | 0.447 | 0.65584 | 0.9998 | 0.197 | -0.645 | 1.039 |
| Day36 | 5xFAD Aged vs WT Aged | -3.714 | -6.584 | -0.844 | 148.1 | -3.58 | 0.00047 | 0.00372 | -1.474 | -2.362 | -0.587 |
| Day36 | 5xFAD Young vs WT Young | 0.413 | -3.167 | 3.993 | 148.2 | 0.319 | 0.75 | 0.99998 | 0.161 | -0.798 | 1.119 |
| Day55 | 5xFAD Aged vs WT Aged | -1.349 | -4.357 | 1.658 | 148.3 | -1.241 | 0.21649 | 0.85798 | -0.534 | -1.366 | 0.299 |
| Day55 | 5xFAD Young vs WT Young | 1.38 | -2.2 | 4.96 | 148.2 | 1.066 | 0.28809 | 0.93403 | 0.536 | -0.438 | 1.511 |
| Day77 | 5xFAD Aged vs WT Aged | 0.723 | -2.284 | 3.731 | 148.3 | 0.665 | 0.50687 | 0.9965 | 0.286 | -0.536 | 1.108 |
| Day77 | 5xFAD Young vs WT Young | 2.047 | -1.534 | 5.627 | 148.2 | 1.581 | 0.11595 | 0.62693 | 0.795 | -0.2 | 1.791 |

**Supplementary Table S1.** Between-group comparisons of intraocular pressure (IOP) between 5xFAD and age-matched wild-type mice at each study time point.

Abbreviations: CI, confidence interval; df, degrees of freedom. Mean differences were calculated as 5xFAD minus age-matched WT values. P values were adjusted using the Sidak method.
